# Clinical Utility of Non‐Invasive Tests for Liver Fibrosis in People Living With Alpha‐1 Antitrypsin Deficiency

**DOI:** 10.1111/liv.70165

**Published:** 2025-06-06

**Authors:** Joost Boeckmans, Jörn M. Schattenberg, Malin Fromme, Pavel Strnad, Hannes Hagström

**Affiliations:** ^1^ Department of Medicine Huddinge, Karolinska Institutet Stockholm Sweden; ^2^ In Vitro Liver Disease Modelling Team, In Vitro Toxicology and Dermato‐Cosmetology, Faculty of Medicine and Pharmacy Vrije Universiteit Brussel Brussels Belgium; ^3^ Department of Medicine II University Medical Center Homburg, Homburg and Saarland University Saarbrücken Germany; ^4^ Medical Clinic III, Gastroenterology, Metabolic Diseases and Intensive Care, University Hospital RWTH Aachen, Health Care Provider of the European Reference Network on Rare Liver Disorders (ERN RARE LIVER) Aachen Germany; ^5^ Division of Hepatology, Department of Upper GI Karolinska University Hospital Stockholm Sweden

**Keywords:** alpha‐1 antitrypsin deficiency, liver cirrhosis, liver stiffness, non‐invasive test, Pi*ZZ, vibration‐controlled transient elastography

## Abstract

Severe alpha‐1 antitrypsin deficiency (AATD) is a rare genetic condition characterised by low systemic levels of alpha‐1 antitrypsin due to its retention in the liver. Consequently, it predisposes individuals to the development of chronic obstructive pulmonary disease and liver cirrhosis. Much progress has been made to non‐invasively monitor liver fibrosis and cirrhosis in individuals with other liver diseases, but it remains unclear how to assess liver disease in people with AATD. This narrative review examined the available evidence on non‐invasive tests (NITs) to stage liver fibrosis and predict incident major adverse liver outcomes in people with AATD. Liver stiffness measurement (LSM) using vibration‐controlled transient elastography (VCTE), blood‐based NITs, and serum liver enzymes are generally normal or mildly elevated in individuals with AATD. Further, VCTE‐LSM and blood‐based NITs, including the AST to platelet ratio index and fibrosis‐4 score, have diagnostic utility for predicting F2 and F3 fibrosis and hold excellent (AUROC ≥ 0.90) prognostic value for incident major adverse liver outcomes. Gamma‐glutamyl transferase also exhibits diagnostic and prognostic utility but is subject to multiple non‐AATD‐related fluctuations. A potential strategy to non‐invasively assess liver disease stage and estimate the risk of major adverse liver outcomes in people with AATD could consist of a combination of VCTE‐LSM with blood‐based biomarker panels. Future studies should explore if liver stiffness naturally fluctuates over time in people with AATD, assess the ideal frequency of follow‐up, and evaluate if NITs can guide the treatment of AATD‐related liver disease.

**Summary**

- Vibration‐controlled transient elastography (VCTE) liver stiffness measurement (LSM) and blood‐based biomarker panels can predict F2/F3 fibrosis in people with alpha‐1 antitrypsin deficiency (AATD)
- VCTE‐LSM best predicts adverse liver outcomes, but the AST to platelet ratio index and fibrosis‐4 score also have prognostic utility.
- Non‐invasive tests for fibrosis have similar or higher discrimination in people with AATD compared to metabolic dysfunction‐associated steatotic liver disease to stage fibrosis and predict adverse liver outcomes, but cutoffs are less established.
- Monitoring of liver disease in people with AATD could be done using a combination of VCTE‐LSM and blood‐based biomarker panels.

**Abbreviations**
AAT: alpha‐1 antitrypsin
AATD: alpha‐1 antitrypsin deficiency
ALT: alanine aminotransferase
APRI: AST to platelet ratio index
ARFI: acoustic radiation force impulse
AST: aspartate aminotransferase
AUROC: area under the receiver operating characteristic
FIB‐4: fibrosis‐4 score
GGT: gamma‐glutamyl transferase
LSM: liver stiffness measurement
MALO: major adverse liver outcome
MASLD: metabolic dysfunction‐associated steatotic liver disease
MRE: magnetic resonance elastography
NFS: NAFLD fibrosis score
NIT: non‐invasive test
Pi: protease inhibitor
SWE: shear wave elastography
VCTE: vibration‐controlled transient elastography

## 1 Introduction

Alpha‐1 antitrypsin deficiency (AATD) is an inherited autosomal codominant condition caused by mutations in the *SERPINA1* gene [1]. AATD predisposes primarily to chronic obstructive pulmonary disease because of excess unopposed neutrophil elastase activity, and to liver cirrhosis due to a toxic gain‐of‐function of alpha‐1 antitrypsin (AAT) in the liver. Lung dysfunction usually develops not earlier than the 3rd or 4th decade of life, while hepatic manifestations present in two peaks in severe AATD, being in early childhood and between the 5th and 6th decade of life, with further increase of incidence [2, 3, 4, 5].

**FIGURE 1:**
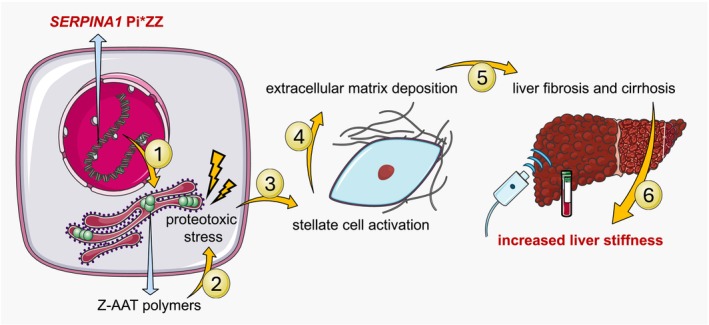
Pathophysiological process resulting in increased liver stiffness in people living with alpha‐1 antitrypsin deficiency due to Pi*ZZ. (1) Homozygous carriage of the Pi*Z variant allele of *SERPINA1*. (2) Aberrant mutant Z‐alpha‐1 antitrypsin forms polymers and accumulates in the rough endoplasmic reticulum of hepatocytes, resulting in proteotoxic stress. (3) Through a process of cell stress, inflammation, and cell death, stellate cells become activated. (4) Activated stellate cells produce excess extracellular matrix. (5) Pathological amounts of extracellular matrix result in liver fibrogenesis and cirrhosis development. (6) Increased liver stiffness can be measured as a consequence of the Pi*ZZ genotype. [abbreviation: Z‐AAT, Z‐alpha‐1 antitrypsin].

There exists variability in disease onset, severity, and presentation, depending on the specific alleles underlying the disease, of which more than 200 variants are known, in combination with environmental factors [6, 7, 8]. The prevalence of AATD differs considerably by geographic region but has been best studied in Europe [9]. One in 25 persons of European descent carries one Z allele (Glu342Lys) instead of two wild‐type M alleles, which culminates in homozygosity, coined the protease inhibitor (Pi)*ZZ genotype, in 1 out of 2000 persons, which accounts for 95% of severe AATD cases [1, 10]. Alpha‐1 antitrypsin is primarily synthesised by hepatocytes, and carriage of the Z allele results in a misfolded variant, named Z‐alpha‐1 antitrypsin (Z‐AAT). Conformational instability of Z‐AAT causes polymer formation in the endoplasmic reticulum and hence impaired secretion into the bloodstream, which is most pronounced upon homozygous Pi*Z carriage [1, 11]. Following intracellular retention, Z‐AAT polymers induce proteotoxic stress, inflammation, and cellular damage, potentially culminating in advanced liver disease, including cirrhosis and hepatocellular carcinoma (Figure 1) [10, 12]. The Pi*S genotype (Glu264Val) has similar or even greater penetration in the population depending on geographic region, but causes less to no severe liver disease since it does not cause polymer formation, unless when accompanied by a Z allele [13]. Further, the Pi*SZ genotype has been reported to affect 1 in 483 subjects in Southern Europe, making it a relatively prevalent subtype in certain regions [14].

Liver‐related complications thus mainly occur in Pi*ZZ carriers, which can be considered a disease‐causing genotype, while heterozygous Pi*Z carriage is a disease‐modifying entity requiring additional hits to cause liver disease [15], such as cystic fibrosis, viral infections, alcohol‐related liver disease, or metabolic dysfunction‐associated steatotic liver disease (MASLD) [16, 17, 18]. After lung damage, liver damage adds to disease‐specific mortality in people living with AATD due to Pi*ZZ [19, 20]. In a Swedish registry‐based cohort with 2286 individuals with AATD without genotyping, the 5‐year cumulative incidences of lung‐ and liver‐related mortality were 3.14% and 1.42% (compared to 0.30% and 0.08% in the general population) [21], respectively, highlighting the need for timely diagnosis and follow‐up of both lung and liver disease in AATD.

Apart from traditional AATD genotypes, also more rare variants, which are prone to misdiagnosis, including Pi*Mmalton and Pi*Siiyama, have been associated with the development of liver disease [22, 23].

While non‐invasive tools are frequently used in the clinical routine for disease staging, liver biopsy is the reference standard to determine AATD‐related liver involvement and should be considered when non‐invasive evaluation of liver disease remains inconclusive in individuals with AATD [20, 24]. However, sparse data is available on how liver involvement and liver disease progression should be tested and monitored in people living with AATD, as also stated in the clinical guideline from the European Association for the Study of the Liver [24]. Therefore, a subset of patients still undergoes liver biopsy as part of the diagnostic work‐up, which is associated with a risk of bleeding and is subject to inter‐observer variability [25].

The aim of this narrative review is to integrate the available evidence on the use of non‐invasive tests (NITs) for liver fibrosis in adults living with AATD and discuss potential strategies to non‐invasively assess and monitor AATD‐related liver disease. Where possible, we make a comparison to the clinical utility of these NITs in MASLD, since much advancement has been made in this field regarding non‐invasive assessment of liver disease [26].

## 2 Search Strategy

Research papers were retrieved from PubMed and Web of Science until 14 March 2025 using search terms (“alpha 1‐Antitrypsin Deficiency”[Mesh] OR “alpha 1‐Antitrypsin Deficiency” OR “AATD” OR “A1ATD”) AND (“non‐invasive test” OR “vibration‐controlled transient elastography” OR “transient elastography” OR “VCTE” OR “fibroscan” OR “elastography” OR “liver stiffness” OR “FIB‐4” OR “fibrosis‐4” OR “APRI” OR “AST to platelet ratio index” OR “NAFLD fibrosis score” OR “NFS” OR “serum test”).

## 3 Natural History and Progression of Alpha‐1 Antitrypsin Deficiency‐Related Liver Disease

AATD, due to the Pi*ZZ genotype, is one of the most common genetic causes of liver disease in newborns and children [27], and a Spanish study found that AATD was the second most frequent reason for paediatric liver transplantation, after biliary atresia [28]. Carriage of the Pi*ZZ variant does not necessarily result in overt liver disease in all infants. While elevated liver function tests are common at birth, only ~10% develop neonatal cholestasis, and even fewer develop a life‐threatening paediatric liver disease [2, 29]. Thus, most infants carrying the Pi*ZZ genotype are asymptomatic, and relevant cues to consider a liver work‐up in the paediatric population include persistently elevated bilirubin and transaminases, prolonged jaundice, signs and symptoms of cirrhosis, and parents known with AATD [13].

In adults, Pi*ZZ‐related liver disease can manifest at any moment but generally remains silent until approximately the age of 50 years, after which symptoms and signs of advanced liver disease, including jaundice and hepatic decompensation, may become apparent. It is estimated that approximately 20%–35% of adults living with AATD due to the Pi*ZZ genotype will develop at least stage two (out of four) liver fibrosis [20, 24], and approximately 15% of adults who present with AATD‐related liver disease require a liver transplantation [30], making this a relevant group for close follow‐up [20, 31]. Notably, liver and lung disease develop independently, and ~50% of subjects with significant liver fibrosis have largely unaffected lung function [12, 32].

Based on the UK Biobank, homozygous carriage of the Pi*Z allele typically confers the highest risk for liver fibrosis or cirrhosis (adjusted odds ratio 21.7, 95% CI = 8.8–53.7) and primary liver cancer (adjusted odds ratio 44.5, 95% CI = 10.8–183.6) [33], which is also supported by other sources [24, 34, 35].

**FIGURE 2:**
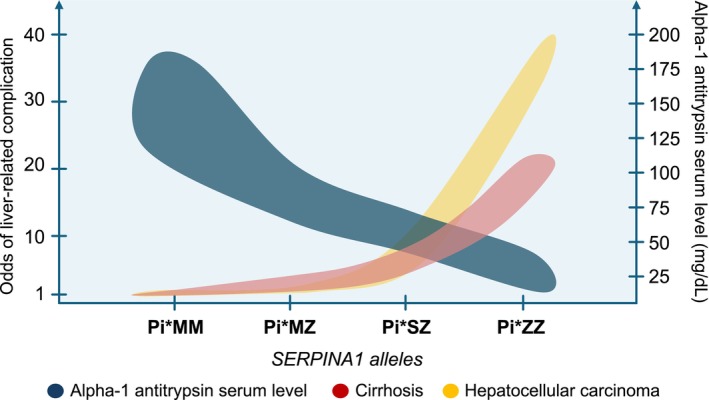
Risk of liver cirrhosis and hepatocellular carcinoma in relation to *SERPINA1* alleles and serum alpha‐1 antitrypsin levels.

The risk to progress from F3 fibrosis to cirrhosis within 5 years was found to be ~55% in a retrospective study including 316 Pi*ZZ individuals (of which 33 with F3 fibrosis) with fibrosis staging using a hierarchical combination of liver biopsy, magnetic resonance elastography (MRE), vibration‐controlled transient elastography (VCTE)‐liver stiffness measurement (LSM), fibrosis‐4 score (FIB‐4), and aspartate aminotransferase (AST) to platelet ratio index (APRI), suggesting a high rate of disease progression [36], although there was a risk of misclassification bias for fibrosis stage. The relative risk for ever developing liver cirrhosis and hepatocellular carcinoma in adults with AATD based on different Pi genotypes is provided in Figure 2 [24, 33].

Risk factors for having fibrosis or cirrhosis in people with AATD were investigated in the UK Biobank in 17 006 Pi*MZ and 864 Pi*SZ carriers, and included age above 50 years (odds ratio in Pi*MZ 1.6, 95% CI = 1.2–2.2, odds ratio in Pi*SZ 2.9, 95% CI = 1.1–7.8), obesity (odds ratio in both Pi*MZ and Pi*SZ 3.9, 95% CI = 1.2–12.2), and male sex (odds ratio in Pi*MZ 1.8, 95% CI = 1.3–2.7; odds ratio in Pi*SZ 3.3, 95% CI = 1.1–10.3) [33], indicating that risk factors for developing advanced liver disease in AATD are similar to other chronic liver diseases. In a Swedish cohort of 1595 Pi*ZZ individuals with mean follow‐up of 12 years, male sex (relative risk 1.45, 95% CI = 1.15–2.14) and age above 50 years (relative risk 2.02, 95% CI = 1.30–3.16) were confirmed as risk factors for liver disease development, which was also true for diabetes mellitus (relative risk 3.87, 95% CI = 2.18–6.87), hepatitis virus infection (relative risk 3.12, 95% CI = 1.21–8.08) and chronic obstructive pulmonary disease (relative risk 2.20, 95% CI = 1.32–3.70) [31].

## 4 Non‐Invasive Tests for Liver Fibrosis in People Living With Alpha‐1 Antitrypsin Deficiency

### 4.1 Serum Liver Enzymes and Blood‐Based Biomarker Panels

Data on specific blood‐based NITs for AATD‐related liver disease are lacking, but several cohort studies have reported on alterations in standard laboratory liver tests and blood‐based biomarker panels for liver fibrosis. The potential utility of standard serum liver tests was investigated in a large European cohort that compared liver enzyme serum levels between Pi*ZZ carriers and non‐Pi*Z carriers. Using an exploratory cohort of 403 Pi*ZZ carriers and 234 controls without any Z allele, mean levels of liver serum enzymes, expressed as a percent of the upper limit of normal, were higher in individuals with the Pi*ZZ genotype compared to those without a Z allele (80% vs. 66% for alanine aminotransferase (ALT), 74% vs. 62% for AST, and 100% vs. 58% for gamma‐glutamyl transferase (GGT) of ULN). Platelet count was also significantly lower in individuals carrying the Pi*ZZ genotype [12], suggesting involvement of patients with cirrhosis with portal hypertension [37]. Consequently, APRI was also significantly higher in Pi*ZZ individuals, and the adjusted odds of having an APRI score of at least 1 (i.e., suggestive of advanced fibrosis) when having the Pi*ZZ genotype was 9.5 (95% CI = 1.2–76.6), where the wide confidence interval should be noted. In the same analysis, carriage of the Pi*ZZ genotype was associated with a 14.8 (95% CI = 5.4–40.4) higher chance of having a HepaScore (which includes age, sex, a2‐macroglobulin, hyaluronic acid, bilirubin, and GGT) of at least 0.72, which is also suggestive of advanced fibrosis. Although serum liver test disturbances are more common in people living with AATD [12], fibrosis stage was not directly assessed, and there was no longitudinal follow‐up to capture incident cases of cirrhosis.

A Swedish registry‐based longitudinal cohort study, including 1595 Pi*ZZ individuals and a mean follow‐up of 12 years, assessed the utility of serum liver tests to differentiate between subjects with (*n* = 155) and without (*n* = 1440) liver disease based on a broad composite definition with International Classification of Disease codes. Aspartate aminotransferase, ALT, and GGT levels were significantly higher at baseline in subjects who developed liver disease compared to those without, with the exception of ALT levels in women, which were comparable between groups. A multivariable analysis showed that repeated elevated liver serum test results, defined as repeated elevation of two or more liver enzymes, were associated with more frequent development of liver disease (relative risk 7.66, 95% CI = 5.10–11.73) [31], suggesting that standard laboratory liver tests could assist in estimating liver disease development.

A smaller, but biopsy‐controlled, study including 94 adults with the Pi*ZZ genotype investigated whether liver enzymes (AST, ALT, GGT), blood‐based scores (FIB‐4 and APRI), and VCTE‐LSM could discriminate significant fibrosis (≥ F2), which was histologically confirmed in 35% of the study participants. It was found that GGT had the highest discriminative capacity (area under the receiver operating characteristic (AUROC) 0.77, 95% CI = 0.67–0.86) when compared to the other blood‐based markers (AUROC FIB‐4, 0.66, 95% CI = 0.54–0.78; APRI, 0.70, 95% CI = 0.58–0.82) and VCTE‐LSM (Table 1). A clear limitation was the exclusion of patients with liver cirrhosis and the limited number of patients with advanced fibrosis (F3 *n* = 6) [32], and a later biopsy‐controlled study showed that GGT levels were normal in 38% of Pi*ZZ individuals with advanced fibrosis (≥ F3) [43].

**TABLE 1:** Comparison of diagnostic and prognostic performances of non‐invasive tests for liver fibrosis between alpha‐1 antitrypsin deficiency and metabolic‐dysfunction associated steatotic liver disease.

Test	Alpha‐1 antitrypsin deficiency (Pi*ZZ)						MASLD/MASH					
	AUROC (95% CI)	*N*	Cutoff	Sens (%)	Spec (%)	Ref.	AUROC (95% CI)	*N*	Cutoff	Sens (%)	Spec (%)	Ref.
≥ *F2 fibrosis (histology)*												
APRI	0.70 (0.58–0.82)	94	0.43	59	87	[32]	0.66 (0.61–0.71)	514	0.42	57	67	[38]
FIB‐4	0.66 (0.54–0.78)	94	1.43	74	64	[32]	0.71 (0.66–0.75)	514	1.12	71	62	[38]
VCTE‐LSM	0.69 (0.58–0.82)	94	5.45 kPa	75	62	[32]	0.77 (0.72–0.82)	373	8.2 kPa	71	70	[39]
≥ *F3 fibrosis (histology)*												
APRI	0.75 (0.52–0.98)	94	0.63	57	94	[32]	0.70 (0.69–0.72)	5477	0.49	67	63	[40]
FIB‐4	0.75 (0.53–0.97)	94	1.90	71	78	[32]	0.76 (0.74–0.77)	5393	1.44	69	70	[40]
VCTE‐LSM	0.92 (0.82–1.00)	94	8.45 kPa	83	89	[32]	0.85 (0.84–0.86)	5489	9.1 kPa	77	78	[40]

Clinical outcomes										
Test	AUROC	*N*	Follow‐up time	Outcome definition	Ref.	AUROC	*N*	Follow‐up time	Outcome definition	Ref.
APRI	0.92	737	5 years	Liver‐related mortality, liver transplantation, or decompensation of liver cirrhosis	[41]	0.80 (0.73–0.86)	320	9 years (median)	Liver‐related events	[42]
FIB‐4	0.90	737	5 years		[41]	0.74 (0.64–0.82)	1032	5 years	All‐cause mortality, hepatocellular carcinoma, liver transplantation, or cirrhosis complications	[26]
VCTE‐LSM	0.95	737	5 years		[41]	0.76 (0.70–0.83)	1193	5 years		[26]

Abbreviations: APRI, AST to platelet ratio index; AUROC, area under the receiver operating characteristic; CI, confidence interval; FIB‐4, fibrosis‐4 score; MASH, metabolic dysfunction‐associated steatohepatitis; MASLD, metabolic dysfunction‐associated steatotic liver disease; sens, sensitivity; spec, specificity, VCTE‐LSM, vibration‐controlled transient elastography—liver stiffness measurement.

The usefulness of GGT for predicting major adverse liver outcomes (MALOs) (liver transplantation, first decompensation of liver cirrhosis, or death due to liver disease) was demonstrated in a prospective multi‐center international cohort study including 737 Pi*ZZ individuals without known liver disease. GGT serum values were elevated at baseline above the upper limit of normal in 89.5% of participants that developed a hepatic endpoint (*n* = 41), compared to 21.7% in those not reaching an endpoint and showed good prognostic performance (AUROC 0.95 and 0.90 after 3 and 5 years; subdistribution hazard ratio for GGT ≥ ULN: 26.7, 95% CI = 9.5–75.4, univariable with non‐liver‐related death considered a competing risk). In the same cohort, the FIB‐4 and APRI also appeared to be useful for predicting MALOs at 3 and 5 years (AUROC FIB‐4 0.93 and 0.90; APRI 0.95 and 0.92) (subdistribution hazard ratio: FIB‐4 ≥ 2.67: 29.2, 95% CI = 15.1–56.1; APRI ≥ 1.0: 45.7, 95% CI = 24.7–84.8) [41]. However, when considering 7.1 kPa determined by VCTE‐LSM as a surrogate for significant fibrosis and 10 kPa for advanced fibrosis, the FIB‐4, APRI, and NAFLD fibrosis score (NFS) showed modest ability for predicting these fibrosis stages (AUROC for LSM > 7.1 kPa: FIB‐4 0.74; APRI 0.72; NFS 0.73; LSM > 10 kPa: FIB‐4 0.68; APRI 0.76; NFS 0.74) in a prospective (non‐peer‐reviewed) study with 155 individuals with different genotypes underlying AATD [44], suggesting that monitoring liver disease progression in lower fibrosis stages solely using blood‐based biomarker panels may be insufficient. When compared to MASLD, APRI and FIB‐4 seem to perform similarly for fibrosis staging but outperform for predicting MALOs in patients with the Pi*ZZ genotype (Table 1). It is also noteworthy that GGT adds significantly to the FIB‐4 in predicting MALOs in the general population [45].

### 4.2 Vibration‐Controlled Transient Elastography

Liver stiffness measurement primarily through VCTE has emerged as a valuable tool for assessing liver fibrosis in different liver diseases [46]. In a cohort including 431 Pi*ZZ adults, it was shown that LSM positively but modestly correlated with circulating Z‐polymer levels (Spearman's rho 0.21, 95% CI = 0.11–0.31) [47], suggesting that liver stiffness, to some extent, relates to specific pathophysiological mechanisms in AATD [1]. Further, in Pi*ZZ carriers, liver enzymes can be in the normal range while having an LSM suggestive of significant fibrosis, and *vice versa* [48], which emphasises the need for liver‐specific methods for detecting liver disease.

Using a liver biopsy‐controlled study sample of 94 Pi*ZZ patients, LSM had an AUROC of 0.69 (95% CI = 0.58–0.82) to differentiate ≥ F2 fibrosis from F0–F1 and appeared to be superior for differentiating ≥ F3 from ≤ F2 fibrosis (AUROC 0.92, 95% CI = 0.82–1.00) compared to blood‐based liver parameters (Table 1) [32]. This is similar to or better than the performance of VCTE in MASLD [40], which could be caused by the lower body mass index and hence smaller distance from the probe to the liver in the Pi*ZZ population compared to people living with MASLD, reducing the risk for measurement error [41, 49]. The best estimated cutoff for significant fibrosis (≥ F2) was 5.45 kPa, compared to 8.45 kPa for advanced fibrosis (F ≥ 3), which performed well to exclude F ≥ 3 with a negative predictive value of 98% [32]. In a smaller German study including adult participants with the Pi*ZZ genotype, subjects were biopsied when having an LSM value of at least 7.1 kPa or recurrent elevated liver enzymes (*n* = 27). From the 23 participants that had an LSM ≥ 7.1 kPa, 22 had at least F2 fibrosis, and only 2 individuals with F3 fibrosis had an LSM below 7.4 kPa, suggesting that a cutoff around 7.1 kPa would perform well to exclude advanced fibrosis [43], which fairly corresponds to the 8 kPa cutoff that is frequently used for risk assessment for advanced liver disease in patients with MASLD [50].

In line with higher cutoffs, carriers of the Pi*ZZ genotype (*n* = 403) had an adjusted 19.8 (95% CI = 4.6–84.1) times higher chance of having an LSM of at least 10 kPa compared to non‐Pi*Z carriers (*n* = 243) (1.3% in non‐Pi*Z carriers compared to 13.6% in Pi*ZZ) [12], suggesting a high risk of MALOs when extrapolating to MASLD data [51].

When trying to predict the risk for incident MALOs using LSM at baseline in 737 Pi*ZZ individuals and a median follow‐up of 3.5 years, LSM was significantly higher in participants that developed an outcome (*n* = 41, 23.6 kPa at baseline, suggestive of cirrhosis (95% CI = 12.9–38.0)), compared to individuals with liver endpoint‐free survival (5.3 kPa (95% CI = 4.3–6.8)). VCTE‐LSM showed excellent prognostic utility for incident MALOs with an AUROC of 0.98 at 3 years and 0.95 at 5 years in this cohort. The rate of MALO‐free survival was consequently lower in participants with LSM ≥ 15 kPa at baseline, which was retained when using non‐liver‐related death as a competing risk (subdistribution hazard ratio 48.1, 95% CI = 25.3–91.7). When combining LSM ≥ 7.1 kPa and APRI ≥ 0.5, a subdistribution hazard ratio of 75.6 (95% CI = 28.5–201.0) was obtained, indicating complementarity of NITs to predict longitudinal outcomes in people with AATD. Further, out of 540 participants with an LSM < 7.1 kPa, none developed a MALO over more than 1900 years of follow‐up. In line with the 7.1 kPa cutoff, progression to an LSM ≥ 7.1 kPa in participants with an LSM < 7.1 kPa at baseline was investigated in a subsample (*n* = 135) with a median follow‐up of 5 years and sequential VCTE measurements available. Progression to an LSM equal to or higher than 7.1 kPa was rare in participants with an LSM below 7.1 kPa at baseline and was primarily seen in those with preexisting risk factors (12 progressors and 123 non‐progressors). Of note, baseline LSM was higher in participants progressing from an LSM below 7.1 kPa to at least 7.1 kPa (6.1 kPa, 95% CI = 5.1–6.3 vs. 4.6 kPa, 95% CI = 4.1–5.5), as well as APRI and serum transaminases [41], indicating the clinical utility of VCTE‐LSM for monitoring liver disease in Pi*ZZ individuals with the added value of blood‐based tests. In addition, the diagnostic performance for detecting ≥ F3 fibrosis and the prognostic utility for predicting MALOs of VCTE‐LSM in Pi*ZZ patients seem to be similar or higher than for MASLD. Although study groups were smaller for AATD and it is methodologically hard to make direct comparisons due to different study designs, definitions, and underlying prevalence of fibrosis, these data suggest good clinical applicability of VCTE‐LSM in people with AATD due to the Pi*ZZ genotype (Table 2), which was also agreed on in a recent Delphi panel [59]. Nonetheless, the relatively short median follow‐up of 3.5 years in the longitudinal Pi*ZZ cohort limits the knowledge on the long‐term risk prediction using VCTE‐LSM [41].

**TABLE 2:** Advantages and limitations of non‐invasive tests for liver fibrosis in people living with alpha‐1 antitrypsin deficiency.

Test	Advantages	Limitations	Ref.
*Liver enzymes*			
GGT	– Performs better than other serum tests to detect significant fibrosis in a population with mild liver disease – Predictive for liver‐related outcomes – Widely available	– Elevated in obesity, cholestatic liver disease, and upon excessive alcohol consumption – Normal in 38% of people with advanced fibrosis	[32] [43] [52]
AST	– Widely available	– Not liver‐specific	[32] [52]
ALT	– Widely available – Relatively liver‐specific	– Prone to non‐AATD‐related elevation	[32] [52]
*Blood‐based biomarker panels*			
APRI	– Predictive for liver‐related outcomes – Widely available	– Not liver‐specific	[32] [41]
FIB‐4	– Predictive for liver‐related outcomes – Widely available	– Not liver‐specific – The role of age as a confounder on the FIB‐4 is unknown in AATD	[32] [41]
HepaScore	– Relatively liver‐specific	– Scarce data – Non‐liver‐related fluctuation	[12] [53]
*Elastography‐based methods*			
VCTE‐LSM	– Predictive for liver‐related outcomes – Performs well to exclude advanced fibrosis – Increasing availability – Liver‐specific	– Influenced by venous congestion – Inaccurate when having severe obesity – Inaccurate after food consumption – Results obtained with the M‐ and XL‐probe are difficult to compare – Requires operator experience	[12] [32] [33] [43] [41] [49] [54]
MRE	– High diagnostic accuracy – Liver‐specific	– Scarce data – Restricted availability – Influenced by venous congestion – Requires a radiology unit – Costly	[55] [56] [57]
ARFI	– Liver‐specific	– Scarce data – Influenced by venous congestion – Requires operator experience – Limited availability	[56] [58] [54] [57]
2D‐SWE	– Liver‐specific	– Scarce data – Influenced by venous congestion – Requires operator experience – Limited availability	[56] [57]

Abbreviations: AATD, alpha‐1 antitrypsin deficiency; ALT, alanine aminotransferase; APRI, AST to platelet ratio index; ARFI, acoustic radiation force impulse; AST, aspartate aminotransferase; FIB‐4, fibrosis‐4 score; GGT, gamma‐glutamyl transferase; MRE, magnetic resonance elastography; SWE, shear wave elastography; VCTE‐LSM, vibration‐controlled transient elastography—liver stiffness measurement.

A Spanish multi‐center study including 81 homozygous and 67 heterozygous Pi*Z carriers showed that none of the heterozygous carriers had an LSM above 7.5 kPa, compared to 8 (9.9%) in the group with Pi*ZZ [48], suggesting that risk‐stratification pathways should be primarily directed to individuals homozygous for the Pi*Z variant. In contrast, a large multicenter cohort including 586 Pi*ZZ carriers, 239 Pi*SZ carriers, and 279 non‐carriers showed that Pi*SZ carriers were more likely to have significant fibrosis (LSM ≥ 7.1 kPa) compared to non‐carriers (adjusted odds ratio 2.6, 95% CI = 1.1–6.1), while having half the risk compared to homozygous Pi*Z carriage (adjusted odds ratio 0.5, 95% CI = 0.2–0.8) [33]. Similar results were seen in the UK Biobank population, where it was shown that Pi*SZ carriage confers a higher risk of fibrosis or cirrhosis (adjusted odds ratio 3.1, 95% CI = 1.1–8.2) compared to Pi*MM individuals, while Pi*MZ individuals presented only a slightly increased risk (adjusted odds ratio 1.7, 95% CI = 1.2–2.2) [33]. In addition, a retrospective study including 474 Pi*MM and 49 Pi*MZ carriers with compensated cirrhosis at baseline showed that subjects with the Pi*MZ genotype more rapidly developed hepatic decompensation (adjusted hazard ratio 1.81, 95% CI = 1.22–2.69) and required a liver transplant or died from liver disease (adjusted hazard ratio 2.07, 95% CI = 1.21–3.52), also when adjusted for model for end‐stage liver disease score [60].

### 4.3 Other Elastography Methods for Assessing Liver Stiffness

Magnetic resonance elastography has been proven to accurately predict the stage of liver fibrosis in people living with MASLD [61], but its implementation for assessing liver fibrosis in people with AATD is poorly explored. A small prospective study including 9 Pi*ZZ carriers that underwent both MRE and liver biopsy showed an AUROC of 0.90 (*p* < 0.0001) to discriminate the presence of any fibrosis (F ≥ 1) and that a cutoff of 3.0 kPa could be used with 80% sensitivity and 100% specificity [55], which is similar to the cutoff for F ≥ 1 in MASLD (2.65 kPa, sensitivity 69%, specificity 82%) [62]. In another small study including 15 participants with asymptomatic AATD (11 Pi*ZZ, 4 Pi*MZ) MRE, acoustic radiation force impulse (ARFI) quantification, and 2D‐shear wave elastography (2D‐SWE) were explored for their potential correlation, which was confirmed for the different techniques (2D‐SWE/MRE *R* = 0.8587; ARFI quantification/2D‐SWE *R* = 0.7425; and ARFI quantification/MRE *R* = 0.6914) [56]. The correlation between 2D‐SWE and VCTE‐LSM was also calculated in a study sample of 18 Pi*ZZ, 8 Pi*MZ, 2 Pi*SZ, and 1 Pi*ZP‐Lowell carrier, yielding a Spearman's rank correlation coefficient of 0.475 (*p* < 0.05). These early data suggest that 2D‐SWE could be used for assessing liver disease in people living with AATD, although the study sample was small and the median LSM by VCTE was 4.8 kPa, indicating a low prevalence of higher fibrosis stages [63].

In a study employing ARFI quantification, including 29 Pi*ZZ, 12 Pi*SZ, and 42 healthy participants, no differences in shear wave speed were found between subjects with AATD and healthy volunteers, nor between the Pi*ZZ and Pi*SZ groups [58]. Since it is well documented that people living with AATD exhibit a higher risk of liver fibrosis, which increases with the frequency of Pi*Z mutations, it can be questioned whether ARFI quantification could add value to the clinical management of people with AATD. Nonetheless, the study participants were relatively young (mean ages 38–39 years) [64], which could imply that these people did not have relevant liver disease.

In conclusion, the scarcity of data on MRE, 2D‐SWE, and ARFI does not currently allow an evaluation of their utility in predicting F2 or F3 fibrosis and MALOs in people with AATD, and studies in the field are needed.

## 5 Influence of External Risk Factors for Liver Disease and Comorbidities on Non‐Invasive Tests for Liver Fibrosis in People Living With Alpha‐1 Antitrypsin Deficiency

Since accumulating evidence suggests that NITs for liver fibrosis can be useful for the diagnosis of liver fibrosis and predicting the risk of future liver‐related events in patients with severe AATD [41], it is relevant to ascertain which factors can influence these NITs and hence potentially increase the risk for liver‐related outcomes, as for example known for alcohol consumption in patients with MASLD and other liver diseases [65, 66].

In a small cross‐sectional study with 69 AATD individuals carrying variable Pi*alleles, age above 50 years and age at diagnosis above 41 years predicted a FIB‐4 ≥ 1.45 [67], although age is a part of the FIB‐4, making it difficult to tease out the individual effect of other components in FIB‐4 [68]. This applied as well specifically to Pi*Z carriers, for whom the presence of type 2 diabetes mellitus was also associated with an 8 times higher risk of having a VCTE‐LSM equal to or higher than 7.1 kPa [67], suggesting that type 2 diabetes mellitus is a modifier of liver stiffness in persons with AATD, or a “double‐hit” since patients with type 2 diabetes mellitus frequently have MASLD. Further studies are needed to verify this.

Conflicting data exists on the impact of alcohol consumption on liver disease severity and progression [65]. The influence of alcohol consumption on NITs for liver fibrosis in adults with AATD has been explored in 2 large cohorts, one based on the UK Biobank (including 17 145 Pi*MZ, 141 Pi*ZZ subjects, and 425 002 Pi*MM carriers), and one from the European Alpha1 liver consortium (including 561 Pi*ZZ individuals). In the UK Biobank cohort, APRI was significantly more often increased above the 1.0 threshold in both Pi*MZ and Pi*MM individuals when comparing individuals consuming harmful amounts of alcohol to low alcohol consumption or abstinence. Self‐reported alcohol consumption had no clear effect on FIB‐4 levels in both Pi*MZ and Pi*MM individuals. The effect of alcohol consumption on APRI and FIB‐4 in Pi*ZZ carriers was uncertain because of low patient numbers. In the European Alpha1 liver consortium cohort, harmful alcohol consumption appeared to have no relevant effect on the FIB‐4, APRI, or VCTE‐LSM, also when employing carbohydrate‐deficient transferrin 1.7% as a surrogate of recent excessive alcohol consumption. The proportion of patients reporting alcohol consumption in harmful amounts was small, accounting for approximately 1% of all participants [69]. Therefore, the lack of effect in the Pi*ZZ cohort might be due to a small sample size. Also, there are well‐known issues with the underreporting of alcohol consumption, even in patients with known liver disease [70, 71], warranting the need for additional studies.

Several smaller cohorts suggested that the presence of AATD variants may promote the development of fibrosis in viral hepatitis, and because of that, a viral liver infection should be looked for and treated in patients with AATD [18, 72, 73].

Since altered iron metabolism is known to occur in advanced liver disease and patients with hemochromatosis are at risk for liver disease, the effect of *HFE* mutations was explored in a group of 380 Pi*ZZ carriers (246 C282Y/H63D non‐carriers, 24 C282Y heterozygous carriers, 99 H63D heterozygous carriers, 7 H63D homozygous carriers, 4 C282Y/H63D compound heterozygous carriers). No clear effect was found of the common H63D and C282Y *HFE* mutations on LSM, although none of the participants were homozygous for C282Y, so no clear inference on an additive interaction between these genotypes can be made [74].

In terms of disease progression, higher baseline VCTE‐continuous attenuation parameter values were found in Pi*ZZ individuals that progressed from an LSM < 7.1 kPa to an LSM ≥ 7.1 kPa (254 dB/m, 95% CI = 213–297 in non‐progressors (*n* = 123) vs. 318 dB/m, 95% CI = 252–362 in progressors (*n* = 12)) with a minimal follow‐up time of 2 years [41], suggesting that hepatic steatosis could be a risk factor for disease progression in individuals with Pi*ZZ, again supporting a role for an interaction with MASLD. This potential relationship is strengthened by the fact that metabolic syndrome was associated with fibrosis in a cohort of 94 individuals with the Pi*ZZ genotype [32, 75].

## 6 Influence of Treatment on Non‐Invasive Tests for Liver Fibrosis in People Living With Alpha‐1 Antitrypsin Deficiency

There exists no approved pharmacological treatment for AATD‐related liver disease, but intravenous AAT augmentation is an established treatment for AATD‐related lung disease to restore anti‐elastase capacity [76], yet with sparse data on liver‐related endpoints. The impact of intravenous AAT augmentation on NITs for liver fibrosis was recently explored in a cross‐sectional analysis of a multinational cohort of 760 adults with the Pi*ZZ genotype and available liver phenotyping. Of the 344 participants that received AAT augmentation treatment for pulmonary treatment, NITs for liver fibrosis were statistically lower albeit with questionable clinical value compared to non‐augmented individuals (APRI 0.34 compared to 0.38, adjusted *p* < 0.001; and LSM 6.5 vs. 7.2 kPa, adjusted *p* = 0.005), which was reflected by the fibrosis stage on liver histology in a subsample of patients (median fibrosis stage 2.0 (augmented, *n* = 15) vs. 4.0 (non‐augmented, *n* = 35), *p* = 0.006), although exhibiting the same amount of AAT inclusion bodies. Multivariable regression models additionally showed an inverse relation between augmentation status and having an LSM ≥ 7.1 kPa (odds ratio 0.54, 95% CI = 0.35–0.85) and APRI ≥ 0.5 (odds ratio 0.31, 95% CI = 0.18–0.51) [77]. Nonetheless, the study participants did not undergo serial assessments of liver disease, and the underlying mechanism of AAT augmentation treatment to ameliorate liver disease remains vague. In particular, it does not target the Z‐AAT‐gain of function in the liver, but was suggested to have anti‐inflammatory properties [1, 78].

Fazirsiran, a small interfering RNA duplex that promotes the degradation of Z‐AAT mRNA, can potentially be the first drug in the armamentarium for the treatment of AATD‐related liver disease [79, 80]. In a 24‐ and 48‐week phase 2 study with 16 Pi*ZZ participants, Fazirsiran met its primary endpoint to reduce liver Z‐AAT concentrations, which was reflected by reductions in liver enzymes and partly by reduced fibrosis on liver histology. As an exploratory endpoint, liver stiffness was measured using VCTE, showing an overall reduction of 12% from baseline at 24 or 48 weeks [79]. These data suggest that LSM can be a useful NIT for the follow‐up of response to Fazirsiran treatment, although a formal correlation analysis between histological and VCTE‐LSM improvement was not conducted. Further, since liver disease develops only in a minority of people with AATD [30], NITs for assessing liver disease will be essential for guiding Fazirsiran treatment if it is approved.

## 7 Discussion

The natural history of liver disease in people living with Pi*Z‐related AATD remains poorly understood, which complicates the generation of screening algorithms and recommendations for follow‐up [24]. Liver disease is most pronounced in people with homozygous carriage of the Pi*Z allele, but people with heterozygous carriage are not fully protected when compared to non‐carriers [33, 48, 60]. Furthermore, heterozygosity for the Pi*Z allele was found in 6.7% of 315 patients with clinically significant portal hypertension (i.e., a hepatic venous pressure gradient ≥ 10 mmHg) compared to 2.8% of 248 liver donors, resulting in an odds ratio of 2.47 (95% CI = 1.03–5.9) for having clinically significant portal hypertension when carrying a Pi*Z allele [81].

Standard laboratory liver tests, including ALT, AST, and GGT, have been explored for their relationship with liver fibrosis stage and liver‐related outcomes. Although most studies were small and did not include longitudinal outcomes, GGT appeared to perform well for both predicting ≥ F2/F3 fibrosis and MALOs in Pi*ZZ carriers [32, 41]. Nonetheless, GGT is an unspecific parameter and increases in both cholestatic liver disease, obesity, after the introduction of certain medications, and upon excessive alcohol consumption, which complicates its use in clinical practice (Table 2) [52]. The most frequently used blood‐based biomarker panels for liver fibrosis in Pi*ZZ adults are FIB‐4 and APRI. Both scores appeared to have a modest discriminative ability for predicting significant fibrosis and have similar performance to GGT for predicting MALOs [32, 41]. VCTE‐LSM has been increasingly explored in the past years for assessing the potential presence of F2/F3 liver fibrosis in people with Pi*ZZ in several studies, as well as for predicting incident MALOs in a large international study [41]. Although one biopsy‐controlled study reported on a VCTE‐LSM cutoff of 5.45 kPa to discriminate ≥ F2 fibrosis in Pi*ZZ individuals [32], most studies employed a cutoff of 7.1 kPa for predicting significant fibrosis, which was also validated using liver biopsy, and LSM < 7.1 kPa was suggested to be appropriate for ruling out advanced fibrosis [43]. In the largest prospective study with Pi*ZZ participants so far, the LSM 7.1 kPa cutoff performed particularly well for excluding individuals not developing MALOs at 2 years’ follow‐up or longer [41]. However, it remains unclear whether the 7.1 kPa cutoff corresponds to MALO risk estimates in patients with MASLD and if diagnostic algorithms can be used interchangeably [26, 50]. Other elastography‐based methods have been poorly explored in people living with AATD, resulting in a limited potential for their use in clinical practice today (Table 2).

Considering the low prevalence of severe AATD in the general population compared to other liver diseases with a high penetration at the population level [82], particularly MASLD [83], it can be questioned whether screening algorithms using blood‐based NITs otherwise used to pre‐screen for a risk of advanced liver disease in the general population [40] should also be applied to people with Pi*Z‐related AATD as a first‐line filter step.

**FIGURE 3:**
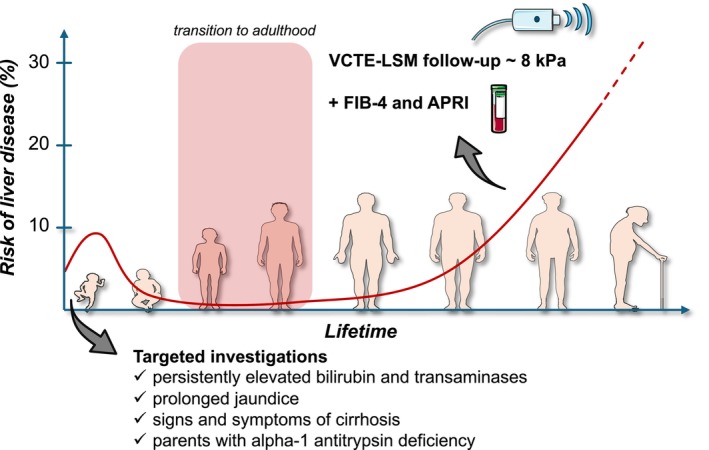
Potential strategy for liver disease follow‐up in people living with alpha‐1 antitrypsin deficiency (Pi*ZZ). Paediatric liver disease becomes apparent in early childhood, requiring no specific screening strategy, but instead targeted investigations based on signs and symptoms of severe liver disease, along with the presence of a potential genetic risk. Through adulthood, periodic liver stiffness measurements using vibration‐controlled transient elastography in combination with blood‐based tests for liver fibrosis seem to hold an adequate balance between sensitivity and specificity for finding severe liver disease and predicting liver‐related outcomes. [abbreviations: APRI, AST to platelet ratio index; FIB‐4, fibrosis‐4 score; LSM, liver stiffness measurement; VCTE, vibration‐controlled transient elastography].

A recent Delphi consensus panel proposed that liver VCTE‐LSM should be applied to individuals with severe AATD, with further workup based on the following LSM categories: LSM < 8 kPa (re‐evaluation in 2–3 years), LSM ≥ 8 –13 kPa (adding MRE and/or ELF, or liver biopsy if discordant results from ≥ 2 NITs, otherwise re‐evaluation in 1–2 years), and LSM ≥ 13 kPa or clear signs of advanced liver disease (MELD/Child‐Pugh score calculation and considering liver transplantation) [59]. Yet, solely relying on VCTE‐LSM results could be error‐prone, as recently observed in patients with type 2 diabetes mellitus, in which a high false‐positive rate for the VCTE‐LSM 8 kPa cutoff was seen [84]. Whether this also applies to people carrying a Pi*Z allele is unknown, but since the FIB‐4 and APRI appear to exhibit a reasonable balance between discriminative utility for F2 and F3 fibrosis and prognostication ability—at least in Pi*ZZ carriers—and availability, a potential strategy for liver disease follow‐up in Pi*Z‐related liver disease could consist of the conjunctive use of these NITs with periodic VCTE‐LSM according to the Delphi consensus depending on regional availability (Figure 3). This way, frequent follow‐up could also partly occur in pulmonary clinics using the FIB‐4 and APRI, with a close connection to specialty liver care using VCTE [41, 59]. The scarcity of data and international consensus on the non‐invasive monitoring of liver disease in people living with AATD due to other Pi‐genotypes than Pi*ZZ leaves a wide gap in current guidelines and recommendations [24]. Since carriers of Pi*MZ and Pi*SZ also exhibit a two‐ and three‐fold risk of fibrosis/cirrhosis, respectively, we advocate liver function tests once a year and VCTE‐LSM every five years, with decision rules as suggested in the recent Delphi consensus for Pi*ZZ carriers, in these patients [33, 59]. Carriers of other Pi‐genotypes could possibly be managed according to serum AAT levels, with patients exhibiting levels below 50 mg/dL being managed according to the consensus on Pi*ZZ‐related AATD, and those with levels above 50 mg/dL being managed similarly to Pi*MZ and Pi*SZ carriers.

## 8 Conclusions and Perspectives

Since LSM through VCTE is a point‐of‐care test and increasingly available [46], regular screening employing LSM with a cutoff of 8 kPa could be a convenient and accurate strategy for finding individuals at risk for liver disease progression, and potentially also select patients that could benefit from liver‐directed treatment [32, 41, 43]. Further studies are needed to examine if cut‐offs used in other fields of hepatology, such as MASLD, implicate the same degree of fibrosis and risk of MALOs. This would likely simplify clinical management. Such a strategy could be used along with frequent determination of the FIB‐4 or APRI to closely monitor potential disease progression.

Although VCTE‐LSM performs well to predict F3 fibrosis, there exists no specific biomarker for liver disease monitoring of patients with AATD, and other more specific biomarkers should be tested, for example, the enhanced liver fibrosis score and pro‐C3. When considering the fair diagnostic performance for detecting F2 and F3 fibrosis by APRI and its prognostic utility for predicting incident MALOs, along with good performance of GGT, although subject to non‐specific fluctuations, a potential specific prediction model for advanced liver disease in patients with AATD could consist of GGT in combination with AST and platelet count. Further, considering the potential impact of the metabolic syndrome and MASLD on the liver disease phenotype in AATD patients, impaired fasting glucose/type 2 diabetes mellitus could be considered as a score component in a more holistic approach. Nonetheless, it should be noted that the causes of death related to MASLD are not interchangeably applicable to Pi*ZZ individuals. This applies in particular to cardiovascular‐related death, which is a leading cause of death in patients with MASLD [85, 86], while respiratory disease and liver disease are the main causes of death in adult Pi*ZZ individuals [21, 87].

It remains to be determined how NITs for liver fibrosis relate to long‐term liver and non‐liver related outcomes in different populations, which factors effectively cause worsening of liver disease, and whether liver fibrosis might regress and fluctuate over time in a natural way. Further, it remains unclear how liver‐targeted treatment of patients with AATD should be guided based on the non‐invasive assessment of liver disease, which will become highly relevant upon approval of liver‐targeted treatments.

## Author Contributions

Conceptualisation: J.B., H.H. Writing – original draft: J.B. Writing – review and editing: J.M.S., M.F., P.S., H.H. Supervision: H.H. All authors approved the final version of the manuscript, including the authorship list.

## Conflicts of Interest

J.B. reports research funding from Colgate‐Palmolive. J.M.S. reports research funding from Gilead Sciences, Boehringer Ingelheim, and Siemens Healthcare GmbH, consulting fees from Apollo Endosurgery, Albireo Pharma Inc., Bayer, BMS, Boehringer Ingelheim, Gilead Sciences, GSK, Intercept Pharmaceuticals, Ipsen, Inventiva Pharma, Madrigal, MSD, Northsea Therapeutics, Novartis, Novo Nordisk, Pfizer, Roche, Sanofi, and Siemens Healthcare GmbH, payment or honoraria from Boehringer Ingelheim, Echosens, MedPublico GmbH, Novo Nordisk, and Madrigal Pharmaceuticals, support for attending meetings and/or travel from Gilead Sciences, and stock from AGED diagnostics and Hepta Bio, outside of the submitted work. M.F. reports consulting fees from Takeda Pharmaceuticals and honoraria from CSL Behring, Grifols Inc., and Takeda Pharmaceuticals. P.S. reports grants and honoraria from Arrowhead Pharmaceuticals, CSL Behring, Grifols Inc., consulting fees or honoraria from AiRNA Pharmaceuticals, Alnylam Pharmaceuticals, Arrowhead Pharmaceuticals, BioMarin Pharmaceutical, Dicerna Pharmaceuticals, GSK, IPSEN Pharmaceuticals, Intellia Pharmaceuticals, SoBi, Takeda Pharmaceuticals, Novo Nordisk, and Ono Pharmaceuticals, participating in leadership or fiduciary roles in Alpha1‐Deutschland, Alpha1 Global, and material transfer support for Vertex Pharmaceuticals and Dicerna Pharmaceuticals. H.H. institutions have received research funding from AstraZeneca, EchoSens, Gilead, Intercept, MSD, Novo Nordisk, and Pfizer. He has served as a consultant or on advisory boards for Astra Zeneca, Bristol Myers‐Squibb, MSD, and Novo Nordisk, and has been part of hepatic events adjudication committees for Arrowhead, Boehringer Ingelheim, KOWA, and GW Pharma.

## Data Availability

Data sharing is not applicable to this article as no new data were created or analysed in this study.
